# DupChecker: a bioconductor package for checking high-throughput genomic data redundancy in meta-analysis

**DOI:** 10.1186/1471-2105-15-323

**Published:** 2014-09-30

**Authors:** Quanhu Sheng, Yu Shyr, Xi Chen

**Affiliations:** Department of Cancer Biology, Vanderbilt University School of Medicine, Nashville, TN 37232 USA; Center for Quantitative Sciences, Vanderbilt University School of Medicine, Nashville, TN 37232 USA; Department of Biostatistics, Vanderbilt University School of Medicine, Nashville, TN 37232 USA

## Abstract

**Background:**

Meta-analysis has become a popular approach for high-throughput genomic data analysis because it often can significantly increase power to detect biological signals or patterns in datasets. However, when using public-available databases for meta-analysis, duplication of samples is an often encountered problem, especially for gene expression data. Not removing duplicates could lead false positive finding, misleading clustering pattern or model over-fitting issue, etc in the subsequent data analysis.

**Results:**

We developed a Bioconductor package Dupchecker that efficiently identifies duplicated samples by generating MD5 fingerprints for raw data. A real data example was demonstrated to show the usage and output of the package.

**Conclusions:**

Researchers may not pay enough attention to checking and removing duplicated samples, and then data contamination could make the results or conclusions from meta-analysis questionable. We suggest applying DupChecker to examine all gene expression data sets before any data analysis step.

**Electronic supplementary material:**

The online version of this article (doi:10.1186/1471-2105-15-323) contains supplementary material, which is available to authorized users.

## Background

Publicly available high-throughput genomic data, especially gene expression data, have greatly changed the way genomic research is conducted recently. The major online databases such as the Gene Expression Omnibus (GEO) [1] ArrayExpress [2], and Sequence Read Archive (SRA) [3] have collected more than one million samples. Not only do these datasets allow the researchers to find relevant individual data set for biomarker validation purpose, multiple data sets can also be combined to increase statistical power to detect the biological patterns that are hidden in one or few datasets with small sample sizes. For example, several studies integrated multiple public available microarray gene expression data to discover new cancer subtypes [4–7]. However, one challenge for gene expression meta-analysis is duplication of samples. In GEO, each individual data set with a unique GEO accession number is associated with a study or publication and was submitted by principal investigator of the study. The gene expression data sets with different accession numbers may contain duplicated samples linked to multiple sample accession numbers. For large-scale gene expression meta-analysis involving hundreds of data sets, the number of duplicated samples may be large.

It is very easy to ignore removal of the duplicated gene expression samples in curated high-throughput data, and the consequences could be obtaining false positive findings or misleading cluster patterns, etc. If the duplicated samples were in both training and testing cohorts for gene signature validation study, it would lead over-fitting of the classifier.

Nevertheless, the identification of duplicated samples could be complicated and labor intensive. For microarray gene expression data, if the preprocessing, normalization and transformation procedures used are different, the normalized datasets for duplicated samples may not be identical. We developed a bioconductor package DupChecker that can efficiently check sample redundancy based on the raw data files of high-throughput genomic data.

## Implementation

The method that we implemented in DupChecker is to examine MD5 hash for each raw data file. MD5 is a message-digest algorithm that can be utilized to check data integrity by producing 128-bit fingerprint of the data input (Rivest, 1992). The duplicated gene expression samples can be identified by checking to see if they have identical MD5 fingerprints.

For users’ convenience, we also developed the functions *geoDownload* and *arrayExpressDownload* to download multiple gene expression data sets from GEO or ArrayExpress databases and deposit the files under the specified directory. The functions *buildFileTable* and *validateFile* will go through each raw data files under the directory to calculate MD5 fingerprint and return a table listing all duplicated samples.

## Result

We applied the DupChecker package to three colon cancer Affymetrix gene expression data sets - GSE13067, GSE14333 and GSE17538 from GEO with 74, 290 and 244 samples respectively. Both GSE13067 and GSE14333 were contributed from the same laboratory in Australia, and GSE17538 was from an institute in the US. The raw data in Affymetrix CEL file format were needed for DupChecker analysis. From the final summary table generated by the *validateFile* function, we found there were 64, 231 and 167 duplicated samples in each data set compared with the other two data sets. Table 1 displays the first few rows of the summary table and the full summary table was listed in Additional file 1. The first column shows the MD5 fingerprint values for the samples with duplications. The rest of the columns are CEL file names for duplications.

**Table 1:** **Illustration of summary table generated by Dupchecker for duplication among GSE13067, GSE14333, and GSE17538 data sets**

MD5	GSE13067(64/74)	GSE14333(231/290)	GSE17538(167/244)
001ddd757f185561c9ff9b4e95563372		GSM358397.CEL	GSM437169.CEL
00b2e2290a924fc2d67b40c097687404		GSM358503.CEL	GSM437210.CEL
012ed9083b8f1b2ae828af44dbab29f0	GSM327335	GSM358620.CEL	
023c4e4f9ebfc09b838a22f2a7bdaa59		GSM358441.CEL	GSM437117.CEL

The DupChecker package is computationally efficient. After downloading CEL files for these three data sets, it took less than one minute to calculate MD5 fingerprints for all 608 files and to deliver a summary table using a 2.7GHz Intel Core i7 CPU.

We also tested DupChecker package on 2 ArrayExpres and 22 GEO breast cancer datasets containing 5203 affymetrix CEL files. Among those files, 696 CEL files were actually duplicated from 348 CEL files. The full summary table and the R code can be found in Additional files 2 and 3. It took around 3 hours on a computer running 64-bit Windows 7 with 2.00GHz Intel® Xeon® E5-2620 CPU and 32.0 GB memory to finish the whole process.

## Conclusions

Gene expression meta-analysis has become increasingly popular for high-throughput genomic data analysis. Due to the large amount of publicly available gene expression data contributed by different researchers, it is almost inevitable to include duplicated samples in the data sets collected for meta-analysis. In the example we showed in Section 3, among the three GEO data sets, there were 463 out of 608 samples with at least one duplicate. It was difficult to tell by IDs since all duplicate samples had different GEO accession numbers. It is effective to identify duplications using MD5 fingerprint generated from raw data since it would not involve any gene expression data processing procedures.

Researchers may not pay enough attention to checking and removing duplicated samples, and then data contamination could make the results or conclusions from meta-analysis questionable. We suggest applying DupChecker to examine all gene expression data sets before any data analysis step. We also want to point out that the data files must be identical to be identified as duplicates. Specimens or RNA samples profiled twice, whether on the sample platform or different platforms, will not be identified using DupChecker.

In this application note, we illustrated the application using gene expression data, but DupChecker package can also be applied to other types of high-throughput genomic data including next-generation sequencing data.

## Availability and requirements

**Project name**: DupChecker

**Project home page**: http://www.bioconductor.org/packages/devel/bioc/html/DupChecker.html

**Programming languages**: R

**Operating system(s):** Platform independent

**Other requirement:** Bioconductor 3.0 or higher

**License:** GPL

## Electronic supplementary material

Additional file 1: **The full result table generated by DupChecker for the colon cancer data.** (CSV 16 KB)

Additional file 2: **The full result table generated by DupChecker for the breast cancer data.** (CSV 28 KB)

Additional file 3: **The R code for breast cancer example.** (PDF 24 KB)

## Abbreviations

GEO: Gene expression omnibus
SRA: Sequence read archive.
