# Supplementary material for: DupChecker: a bioconductor package for checking high-throughput genomic data redundancy in meta-analysis
Source: BMC Bioinformatics. 2014 Sep 30;15(1):323. doi: 10.1186/1471-2105-15-323 (PMC4261523; doi:10.1186/1471-2105-15-323)
Supplement: Supplementary file 3 — Additional file 3: The R code for breast cancer example. (PDF 24 KB) [file 12859_2014_6634_MOESM3_ESM.pdf]

```
library("DupChecker")

rootDir<-"H:/temp/"

starttime<-proc.time()

arrayExpressDownload(c("E-MTAB-365", "E-TABM-158"),  targetDir = rootDir,
overwrite=TRUE, filePattern="cel$")

geoDownload(c("GSE1456", "GSE1561", "GSE2034", "GSE2603", "GSE4922",
"GSE5327", "GSE5460",
            "GSE5462", "GSE5847", "GSE6532", "GSE6772", "GSE7390", "GSE11121",
"GSE12276", "GSE12763", "GSE18864",
            "GSE20194", "GSE20711", "GSE25066", "GSE31448", "GSE31519"),  targetDir
= rootDir, overwrite=TRUE, filePattern="cel$")

datafile<-buildFileTable(rootDir, filePattern="cel$")
result<-validateFile(datafile)
if(result$hasdup){
  duptable<-result$duptable
  write.csv(duptable, file=paste0(rootDir, "/BreastCancerValidataion.csv"))
}

endtime<-proc.time()

cat("Total cost time =", (endtime - starttime)[3], "seconds")
```
